# Herbal medicine use in Thai patients with type 2 diabetes mellitus and its association with glycemic control: A cross-sectional evaluation

**DOI:** 10.1016/j.heliyon.2022.e10790

**Published:** 2022-09-28

**Authors:** A. Prasopthum, T. Insawek, P. Pouyfung

**Affiliations:** a School of Pharmacy, Walailak University, Nakhon Si Thammarat, 80161, Thailand; b Biomass and Oil Palm Center of Excellence, Walailak University, Nakhon Si Thammarat, 80160, Thailand; c School of Public Health, Walailak University, Nakhon Si Thammarat, 80160, Thailand; d Research Center of Workers Health, Walailak University, Nakhon Si Thammarat, 80161, Thailand

**Keywords:** Herbal medicine, Type 2 diabetes mellitus, Medicinal plants, Hemoglobin A1c

## Abstract

**Introduction:**

Herbal medicine has been integrated into Thai culture for many centuries. However, studies on using herbal medicine in combination with antidiabetic agents for glycemic control in managing diabetes are limited. Herein, we aimed to assess the use of herbal medicines with different dosages of antidiabetic agents and their association with glycemic control in Thai patients with type 2 diabetes mellitus (T2DM).

**Methods:**

This hospital-based study included 739 patients with T2DM who consecutively visited four district hospitals in Thailand. An interviewer-administered questionnaire was used to collect patient-specific information, including hemoglobin A1c (HbA1c) levels. Chi-square and logistic regression analyses were used to assess associations and predictors, respectively.

**Results:**

The prevalence of herbal medicine use was 37.5% (n = 264); 70.5% of the patients received information about herbal medicine usage for glycemic control from their relatives and friends, and 21 herbal plants were consumed in combination with their prescribed antidiabetic drugs. The use of herbal medicine was associated with the patients’ educational level (*p* = 0.001), income (*p* < 0.001), and duration of diabetes (*p* < 0.001). Good glycemic control (HbA1c < 7.0%) was associated with the use of bitter gourd in combination with 500 mg/day of the antidiabetic drug metformin (adjusted odds ratio = 8.33, 95% confidence interval = 1.04–66.49, *p* = 0.046). These patients were 2.92 times more likely to have good glycemic control than those who relied solely on 500 mg/day of metformin (adjusted OR = 2.921, 95% CI = 1.227–6.952, *p* = 0.015).

**Conclusions:**

The prevalence of herbal plant use was associated with different variables, including age, BMI, T2DM duration, and metformin dosage. Among the 21 herbal plants, the consumption of bitter gourd with 500 mg/day of metformin was associated with good glycemic control.

## 1 Introduction

Type 2 diabetes mellitus (T2DM), a chronic metabolic disease, has been reported as one of the leading global health problems affecting 9.3% of the world population in 2019 [1]. The International Diabetes Federation estimated that the number of patients with diabetes would reach 642 million by 2040 [1]. In Thailand, the prevalence of diabetes increased from 795 to 1,032 cases/100,000 population from 2010 to 2014. Additionally, the cost of diabetes treatment incurred by patients and the Thai government is significantly rising. Furthermore, the mortality rate of Thai people due to diabetes increased from 10.7 per 100,000 to 17.5 per 100,000 persons from 2010 to 2014 [2].

T2DM pathogenesis is related to insulin resistance, which impairs the ability of insulin target cells (i.e., skeletal muscle and adipose tissue) to absorb and intracellularly metabolize glucose. This results in hyperglycemia and complications, such as neuropathy, age-related macular degeneration, nephropathy, coronary artery disease, and diabetic foot [3, 4]. Patients with T2DM are generally diagnosed based on their fasting blood glucose (>126 mg/dL) and hemoglobin A1c (HbA1c; >6.5%) levels [5]. Although several antidiabetic agents, such as metformin and pioglitazone, are available, successful T2DM management, reflected by good glycemic control, remains challenging. Several factors have been found to affect the efficacy of conventional treatments for T2DM, including adherence to treatment regimens and maintaining a healthy lifestyle, such as regular exercise, healthy eating habits, and maintaining an optimal body weight [6]. Some patients with T2DM also experienced intolerance and adverse side effects with antidiabetic drugs, for instance, in the case of hypoglycemia. Despite the use of conventional medicine, it has been reported that patients with poor glycemic control tend to opt for complementary and alternative medicine (CAM) because of their personal or spiritual beliefs, religion and faith, or recommendations by their family or friends [7, 8, 9, 10]. Patients with diabetes are reportedly 1.6 times more likely to use CAM than those without diabetes [11].

CAM is defined as an array of healthcare systems, practices, and products with a history of use or origins different from conventional Western medicine and is classified into three modalities: 1) use of natural products such as herbs, vitamins, minerals, and probiotics; 2) mind and body practices such as yoga, meditation, massage, and acupuncture; and 3) other complementary health approaches such as naturopathy, Ayurvedic medicine, and traditional Chinese medicine [12]. Natural product-based practices, such as herbal medicine, are the most widely used modalities for CAM among patients with diabetes [12, 13]. Several herbal plants have been used in combination with conventional medicines to treat chronic diseases, including diabetes [7]. For instance, ivy gourd (*Coccinia indica*), bitter gourd (*Momordica charantia*), and cinnamon (*Cinnamomum verum*) have been reported to exhibit glucose-lowering activities both *in vitro* and *in vivo* [7, 9, 14, 15]*.* Reportedly, patients with T2DM tend to use herbal medicine because of concerns about the possible side effects of antidiabetic agents and their personal belief that herbal remedies from natural sources are safer and more effective. However, healthcare professionals have often demonstrated minimal acceptance of herbal medicine to manage diabetes [16, 17, 18]. This could be attributed to the insufficiency of well-designed scientific reports or long-term clinical trial studies on efficiency, possible adverse side effects, and herbal-drug interaction [19].

Although research on the pharmacology of the phytochemical constituents of herbal plants and the prevalence of herbal medicine use among patients with chronic diseases, including diabetes, has been extensively undertaken in Thailand [10, 20, 21, 22], studies on herbal medicine used in combination with prescribed antidiabetic agents for T2DM management in Thai patients in primary care units are rarely documented. Therefore, this study aimed to ascertain the prevalence of herbal medicine used in combination with antidiabetic agents and its association with glycemic control (reflected by HbA1c levels) in patients with T2DM at the primary care clinics of four district hospitals across Thailand. In addition, the sociodemographic information, type, source, frequency, usage purpose, and the form of preparation of herbal supplements that the patients consumed were extensively explored in this study.

## 2 Materials and methods

### 2.1 Study design, sample, and procedure

This cross-sectional study included adults diagnosed with T2DM for at least one year from four district hospitals in Thailand. A district hospital is one of the healthcare services in Thailand that aims to provide primary healthcare services to inpatients (30–120 beds) and outpatients, covering a total population of c.a. 50,000 people. Outpatient care at the district hospital plays a central role in diabetic care by providing patients with T2DM with free medical care, including screening, first treatments (i.e., diet controls or oral antidiabetic agents), and add-on treatments (i.e., in the case of uncontrolled blood glucose) together with a follow-up. In contrast, the province and regional hospitals provide tertiary care for patients with diabetic complications. Monotherapy with 500 mg/day of metformin is the first-line oral antidiabetic agent for diabetes care in Thailand. The metformin dose was gradually increased to the maximum tolerated dose of 2000 mg/day. In the case of severe hyperglycemia, a combination of metformin and other antidiabetic agents (e.g., pioglitazone and sulfonylurea) is used. Patients in our study were recruited during follow-up visits using consecutive sampling. Using consecutive sampling, patients were recruited during their visits for follow-up care or refilling medications from October 11, 2018, to October 10, 2019. Patients with T2DM who had well-recorded diabetes-specific information, including the duration of T2DM, HbA1c levels, types/doses of conventional antidiabetic medication, and were able to participate in face-to-face interviews were included. Pregnant women aged <35 years, patients with incomplete diabetes-specific information, or those who could not complete the individual interviews were excluded. As the total number of patients with T2DM visiting the four primary hospitals was approximately 6,000, the estimated sample size was 375 (calculated using Yamane's formula (N = 6,000, e = 0.05). The sample size was increased to 739 patients to obtain reliable data.

### 2.2 Ethical approval

This study was approved by the Human Research Ethics Committee of Walailak University, Thailand (WU-EC-AH-2081-61) on October 11, 2018. Written informed consent was obtained from all the participants. The questionnaires were anonymized, and patients were free to opt out of participation in the study whenever they were uncomfortable.

### 2.3 Study instrument

All participants provided consent before face-to-face interviews. The questionnaire, comprising three main parts, was adapted and modified from that used in a previous study [23]. The initial draft of the questionnaire was reviewed by healthcare professionals (i.e., doctors and pharmacy academic staff) who commented on the wording and content before the final questionnaire was developed. The content validity index and item objective congruence index of the final questionnaire were 0.92 and 0.82, respectively. The first part of the questionnaire covered the patient's sociodemographic information (sex, age, marital status, educational level, and monthly income) and the duration of T2DM after the first diagnosis. In the second part, we investigated the prevalence of herbal product usage (e.g., type, source, frequency, purpose, and form of preparation). Finally, we retrospectively collected patient-specific data on HbA1c levels and types and doses of prescribed antidiabetic drugs.

### 2.4 Data collection

**Figure 1:**
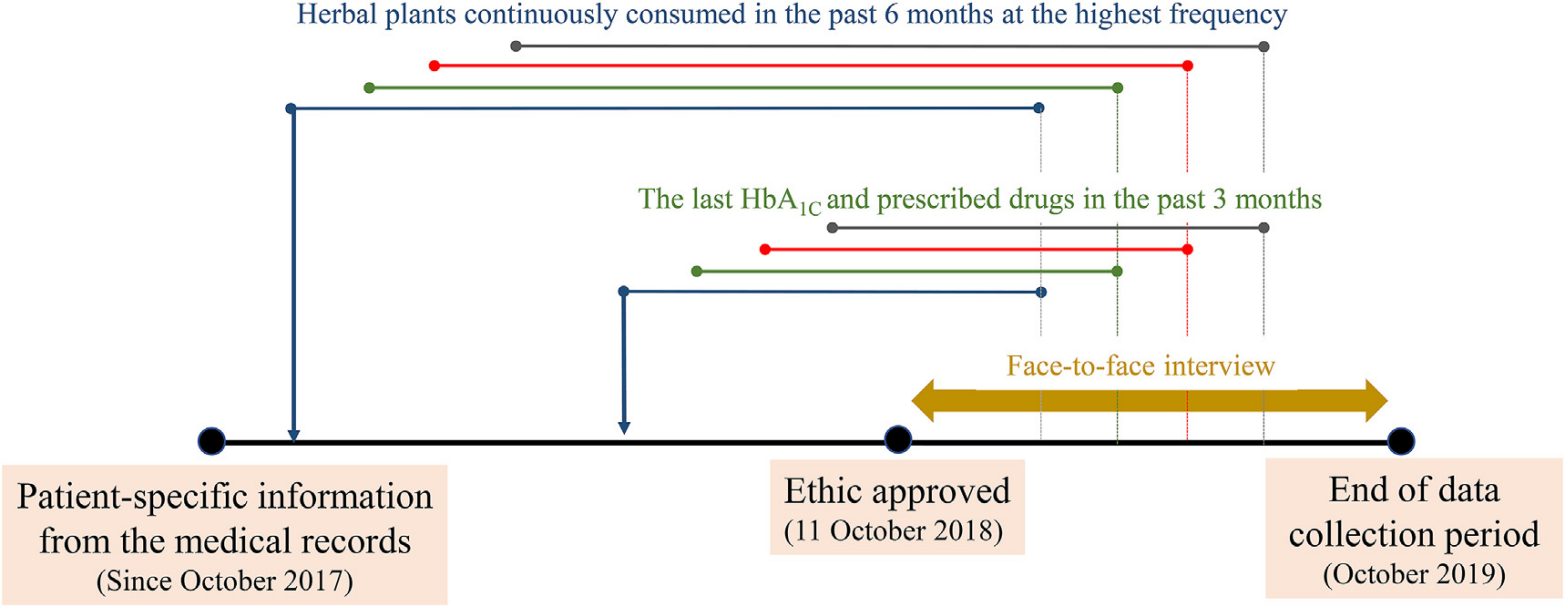
Timeline of data collection, ethical approval, and performing a face-to-face interview.

Data were collected by healthcare professionals trained to interview the participants, measure body mass index (BMI), and evaluate the patient's energy intake and physical activity at the clinic to ensure the reliability and reproducibility of the collected data. BMI (kg/m^2^) was calculated as weight (kg) divided by height squared (m^2^), and physical activity was assessed according to the criteria for the classification of physical activity levels [24]. A herbal plant that the patients had continuously consumed during the past six months at the highest frequency from the date of being interviewed to control their glycemic status was recorded (Figure 1). The local and common names of the plants were matched with their figures and scientific names using the database from www.plantlist.org and were confirmed by the patients. The last HbA1c value in the past three months and the history of being prescribed antidiabetic agents within the previous six months (Figure 1) were obtained from the patient's medical record and cross-verified with the Java Health Center Information System, where patient-specific information was synchronized among different hospitals across Thailand.

### 2.5 Statistical analyses

The collected data were processed and statistically analyzed using R 3.0.2 in RStudio GUI version 1.3.959 (R Foundation for Statistical Computing, Vienna, Austria). Data entered into the software were verified using double-entry verification. The results are reported as frequencies, percentages, or mean ± standard deviations. A chi-square test of independence was used to assess possible relationships between the variables. The association between herbal medicine use and glycemic control was determined using binary logistic regression (crude, model 1). Significant variables (*p* < 0.05) were then used for multivariable analysis (adjusted, model 2), which was performed in the final step to control for the possible confounding effect of the selected variables. Statistical significance was set at *p* < 0.05.

## 3 Results

Sociodemographic characteristics associated with herbal medicine use are shown in Table 1. Of the 739 patients, the majority were female (80.8%), aged ≥60 years (57.9%), married (76.7%), and educated up to the primary school level (81.6%). Approximately one-third of the participants were agriculturists (34.1%), with an income of <9,000 Thai baht/month (<300 USD/month). All patients were diagnosed with T2DM for >1 year, and 56.6% experienced T2DM complications for ≥5 years. Approximately half (53.5%) of the participants were overweight, and 49.6% were prescribed 1000 mg/day of metformin. Most patients (84.8%) had an energy intake of 1201–2400 kcal/day, and 59.9% of the total population participated in light-intensity physical activity according to the criteria for the classification of physical activity levels.

**Table 1:** Sociodemographic characteristics of patients with T2DM (n = 739) and herbal medicine use.

Variables	Overall n (%)	Herbal medicine		*p*-value^a^
		User	Non-user	
**Sex**				
Male	142 (19.2)	54 (7.3)	88 (11.9)	0.524
Female	597 (80.8)	210 (28.4)	387 (52.4)	
**Age (years)**				
30–45	28 (3.7)	4 (0.5)	24 (3.2)	0.007∗
45–60	283 (38.3)	116 (15.7)	167 (22.6)	
>60	428 (57.9)	144 (19.5)	284 (38.4)	
**Educational level**				
Primary school	604 (81.6)	196 (26.5)	408 (55.2)	0.001∗∗
High school	76 (10.3)	44 (6.0)	32 (4.3)	
University	59 (8.0)	24 (3.2)	35 (4.7)	
**Marital status**				
Unmarried	172 (23.3)	52 (7.0)	120 (16.2)	0.086
Married	567 (76.7)	212 (28.7)	355 (48.0)	
**Occupations**				
Government official	39 (5.4)	20 (2.7)	19 (2.6)	<0.001∗∗
Grocer	92 (12.4)	48 (6.5)	44 (6.0)	
Fisherman	144 (19.5)	36 (4.9)	108 (14.6)	
Agriculturist	252 (34.1)	88 (11.9)	164 (22.2)	
Unemployed	212 (28.7)	72 (9.7)	140 (18.9)	
**Average monthly income**				
<9,000 baht	496 (67.1)	162 (21.9)	334 (45.2)	0.013∗
>9,000 baht	243 (32.9)	102 (13.8)	141 (19.1)	
**Duration of T2DM**				
<5 years	321 (43.4)	82 (11.1)	239 (32.3)	<0.001∗∗
5–10 years	262 (35.5)	130 (17.6)	132 (17.9)	
>10 years	156 (21.1)	52 (7.0)	104 (14.1)	
**BMI (kg/m**^**2**^**)**				
18–23 (normal)	176 (23.8)	60 (8.1)	116 (15.7)	0.041∗
23–29 (overweight)	395 (53.5)	156 (21.1)	239 (32.3)	
>30 (obese)	168 (22.7)	48 (6.5)	120 (16.2)	
**Physical activity levels**^b^				
Light intensity	443 (59.9)	144 (19.5)	299 (40.5)	0.001∗∗
Moderate intensity	232 (31.4)	84 (11.4)	148 (20.0)	
Vigorous intensity	64 (8.7)	36 (4.9)	28 (3.8)	
**Dietary intake (kcal/day)**				
<1200	68 (9.2)	28 (3.8)	40 (5.4)	0.002∗∗
1201–1800	340 (46.0)	128 (17.3)	212 (28.7)	
1801–2400	287 (38.8)	104 (14.1)	183 (24.8)	
>2400	44 (6.0)	4 (0.5)	40 (5.4)	
**Oral antidiabetic agents**				
500 mg/day metformin	112 (15.2)	44 (6.0)	68 (9.2)	<0.000∗∗
1000 mg/day metformin	366 (49.6)	126 (17.1)	240 (32.5)	
2000 mg/day metformin + 30 mg/day pioglitazone	261 (35.3)	92 (12.4)	169 (22.8)	

Abbreviations: T2DM, type 2 diabetes mellitus; BMI, body mass index. a Chi-square test. ∗ 0.05 < *p* < 0.001. ∗∗*p* ≤ 0.001. b Classified based on the estimation of energy expenditure by metabolic equivalent (MET) [24].

Of the participants recruited in this study, 35.7% (n = 264) used herbal medicines with prescribed antidiabetic drugs. The chi-square test of independence revealed statistically significant associations between age, educational level, income, BMI, physical activity level, dietary intake, and herbal medicine use (0.001 < *p* < 0.05). Occupation, T2DM duration, and the daily dose of antidiabetic drugs were highly significant factors (*p* < 0.001). There were no associations between sex, marital status, and herbal medicine use (Table 1).

**Figure 2:**
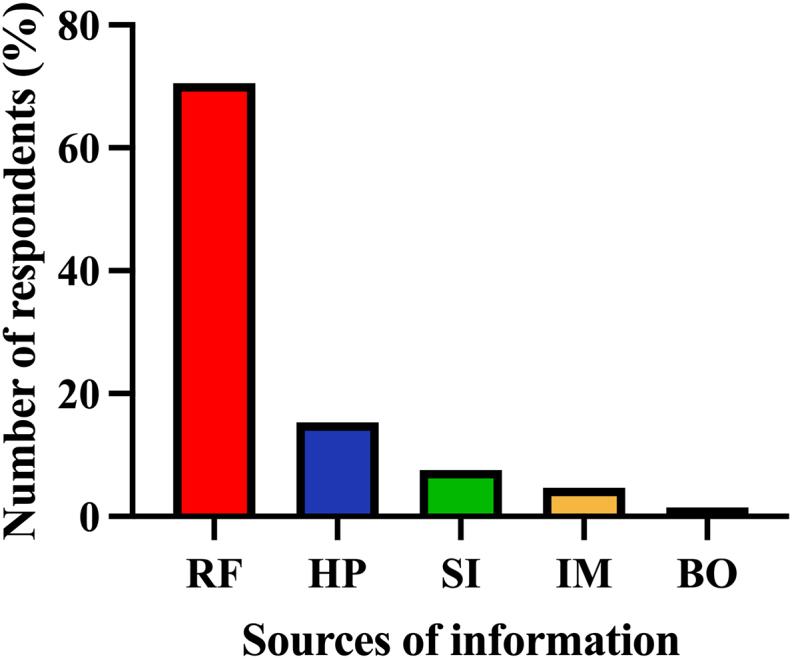
Primary information sources about herbal medicine recommended to patients with T2DM (n = 264). Abbreviations: T2DM, type 2 diabetes mellitus; RF, Relatives and Friends; HP, Healthcare professionals; SI, Self-initiated knowledge; IM, Internet media; BO, Books.

Most herbal medicine users (70.5%) reported receiving preliminary information from their relatives and friends regarding the recommended types of herbal plants for glycemic control. The remaining patients received information from healthcare professionals (15.3%), self-initiated knowledge (7.6%), the internet (4.6%), and Thai traditional medicine books (1.5%) (Figure 2).

There were 21 species of herbal plants that the patients (n = 264) consumed as prescribed antidiabetic drugs (Table 2). The five most commonly reported plants were pandan leaf (*Pandanus amaryllifolius*, 23.1%), bitter gourd (*M. charantia*, 19.2%), country mallow (*Abutilon indicum,* 10.8%), yanang leaf (*Tiliacora triandra*, 6.2%), and lingzhi mushroom (*Ganoderma lucidum*, 6.2%). Different parts of the plants, such as the leaves, dried roots, fruits, and stems, were used to prepare herbal supplements. Tea was the most common form of herbal supplement intake. The other edible forms of herbal supplements consumed by the patients included juices, capsules, and boluses (Table 2).

**Table 2:** Types and parts of herbal plants and forms of preparation with % prevalence.

Common name	Scientific name	Part of use	Supplement Form	%
Pandan leaf	*Pandanus amaryllifolius*	Leaves	Tea	23.1
Bitter gourd	*Momordica charantia*	Fruit	Juice/capsule	19.2
Country mallow	*Abutilon indicum (L.)*	Leaf, twig, root	Tea	10.8
Yanang leaf	*Tiliacora triandra*	Leaves	Tea	6.2
Lingzhi mushroom	*Ganoderma lucidum*	Dried fruiting bodies	Capsule	6.2
Ginger	*Zingiber officinale*	Rhizome (juice)	Tea	5.4
Miscellaneous				
Passion fruit	*Passiflora edulis*	Fruit (juice)	Juice	3.1
Indian marsh fleabane	*Pluchea indica*	Dried leaves	Tea	3.1
Sacha peanut	*Plukenetia volubilis*	Seed	Roasted seed	3.1
Kariyat	*Andrographis paniculata*	Leaves	Tea	3.1
Lime	*Citrus aurantifolia*	Fruit juice	Juice	1.5
Lemongrass	*Cymbopogon citratus*	Leaves	Tea	1.5
Carpet grass	*Axonopus compressus*	Leaves	Tea	1.5
Betel leaf	*Piper sarmentosum*	Leaves	Fresh leaves	1.5
Jewel Vine	*Derris scandens*	Stem	Capsule	1.5
Gurmar	*Gymnema inodorum*	Leaves	Tea	1.5
*Moringa*	*Moringa oleifera*	Leaves	Tea	1.5
Soursop	*Annona muricata*	Leaves	Boiled leaves	1.5
Garlic	*Allium sativum*	Cloves	Fresh cloves	1.5
Kassod	*Senna siamea*	Leaves	Boiled leaves	1.5
Tongkat ali	*Eurycoma longifolia*	Root	Tea/bolus	1.5

The association of herbal medicine usage with good glycemic control, reflected by the HbA1c levels <7.0%, was analyzed using a bivariate regression model against the three major patterns of the antidiabetic drugs prescribed to the patients (500 mg/day metformin, 1000 mg/day metformin, and 2000 mg/day metformin plus 30 mg/day pioglitazone) (Crude (model 1), Table 3). The use of herbal medicine with 500 mg/day of metformin was found to be significantly associated with good glycemic control (odds ratio (OR) = 3.58*,* 95% confidence interval (CI) = 1.58–8.13, *p* = 0.002) and was chosen for multivariable logistic analysis (adjusted (model 2), Table 3), where a significant association was established (adjusted OR = 2.92, 95% CI = 1.22–6.95, *p* = 0.015) with glycemic control.

**Table 3:** Associations between T2DM treatment strategies and glycemic control.

T2DM treatment strategies	Overall	Glycemic control		Crude (model 1)^a^				Adjusted (model 2)^b^			
	N (%)	HbA1c>7%	HbA1c<7%	OR	95% CI		*p*-value	OR	95% CI		*p*-value
500 mg/day metformin											
Non-users	68 (60.7)	39 (34.8)	29 (25.9)	Ref^c^				Ref^c^			
Users	44 (39.3)	12 (10.7)	32 (28.6)	3.58	1.58	8.13	0.002*	2.921	1.227	6.952	0.015*
1000 mg/day metformin											
Non-users	274 (66.5)	182 (44.2)	92 (22.3)	Ref^c^				Ref^c^			
Users	138 (33.5)	98 (23.8)	40 (9.7)	0.80	0.52	1.26	0.346	0.410	0.205	0.818	0.011
2000 mg/day metformin + 30 mg/day pioglitazone											
Non-users	135 (62.8)	96 (44.7)	39 (18.1)	Ref^c^				Ref^c^			
Users	80 (37.2)	60 (27.9)	20 (9.3)	0.82	0.43	1.53	0.537	1.341	0.652	2.760	0.425

T2DM, type 2 diabetes mellitus; HbA1c, hemoglobin A1c; CI, confidence interval; OR, odds ratio. b Model adjusted for sociodemographic characteristics (sex, age, BMI, physical activity levels, dietary intake, and duration of DM).

*p < 0.05.

a Binary logistic regression analysis.

b Multivariable analysis.

c Referenced values.

In Table 4, we analyzed the association of each type of herbal plant with the prescribed 500 mg/day of metformin and glycemic control (Table 4). The five most commonly used herbal plants (Table 2) were selected for the analysis. The use of bitter gourd (*M. charantia*) with 500 mg/day of metformin was associated with good glycemic control in the bivariate analysis (OR = 5.72, 95% CI = 1.36–33.05, *p* = 0.019) and was included in the multivariable analysis. Patients with T2DM who consumed bitter gourd with 500 mg/day of metformin were 8.33 times more likely to have good glycemic control than those who used only 500 mg/day of metformin (adjusted OR = 8.33, 95% CI = 1.04–66.49, *p* = 0.046).

**Table 4:** Associations between T2DM treatment strategies (herbal plants specified) and glycemic control.

T2DM treatment strategies	Glycemic control		Crude (model 1)^a^				Adjusted (model 2)^b^			
	HbA1c > 7%	HbA1c < 7%	OR	95% CI		*p*-value	OR	95% CI		*p*-value
500 mg/day metformin										
Non-users Users of	39 (34.8%)	29 (25.9%)	Ref^c^							
Pandan leaf	4 (3.6%)	8 (7.1%)	2.69	0.74	9.79	0.071	1.29	0.33	5.01	0.711
Bitter gourd	1 (0.9%)	11 (9.8%)	5.72	1.36	33.05	0.019*	8.33	1.04	66.49	0.046*
Country mallow	3 (2.7%)	5 (4.5%)	2.24	0.49	10.14	0.341	0.21	0.02	2.25	0.200
Yanang leaf	2 (1.8%)	2 (1.8%)	1.34	0.18	10.11	0.999	0.64	0.08	5.09	0.677
Lingzi mushroom	2 (1.8%)	2 (1.8%)	1.34	0.19	10.18	0.999	3.45	0.32	36.21	0.302

T2DM, type 2 diabetes mellitus; HbA1c, hemoglobin A1c; CI, confidence interval; OR, odds ratio. b Model adjusted for sociodemographic characteristics (sex, age, BMI, physical activity levels, dietary intake, and duration of D.

* *p* < 0.05.

a Binary logistic regression analysis.

b Multivariable analysis.

c Referenced values.

## 4 Discussion

Several studies have reported increasing trends of herbal medicine use in patients with T2DM; however, studies on herbal medicine usage together with Western medicine in Thai patients are limited. A high prevalence of herbal medicine usage in Thai patients with chronic diseases, not specifically T2DM, has been reported [25]. This study revealed a relatively high prevalence of herbal medicine use (35.7%), specific to Thai patients with T2DM, from four district hospitals in Thailand (n = 739). This is consistent with a recent systematic review and meta-analysis in which a high global prevalence of CAM use among patients with diabetes was reported, and the most popular CAM modality was herbal medicine [26].

Approximately 60% of all patients with T2DM in this study were aged >60 years, the majority of whom were women. Our data agreed with a recent cross-sectional study by Sakboonyarat et al. [27], who also found that the number of female Thai patients with T2DM doubled compared to male patients. Of the female patients, 81.3% had a BMI >23 kg/m^2^ (overweight) and low physical activity. Obesity has been regarded as a risk factor for diabetes, and obese women have a higher risk of developing T2DM than obese men [28]. Our findings showed the associations between different variables and herbal medicine usage among patients with T2DM (including age, BMI, occupation, duration of T2DM, and the daily dose of antidiabetic drugs) (Table 1). Other studies reported similar results wherein age (older), occupation (farming), duration of T2DM (>1 year), having two or more chronic diseases, or even living in rural areas were more likely to influence the use of herbal medicine [29, 30, 31]. Mamo et al. showed that a longer duration of diabetes was associated with poor glycemic control in patients, resulting in persistent hyperglycemia and more severe clinical complications [32]. It has been hypothesized that patients with prolonged diabetes might become unresponsive to antidiabetic drugs or experience adverse side effects from the long-term use of antidiabetic drugs, leading to the decision to include herbal medicine in their T2DM management regimens [33, 34]. In addition, a study of Malaysian patients with T2DM showed that 56% of the patients who used CAM, including herbal medicines, showed good health, and 63.2% of the patients reported high quality and safety of herbal medicine use [35]. The use of herbal medicine was more acceptable to people in low- and middle-income countries; it largely depended on individuals’ spiritual beliefs and traditions in local societies [25, 30, 36]. Easy accessibility, excessive availability, and low cost could contribute to using herbal medicines [29, 35, 37].

The use of herbal plants for glycemic control has been increasingly reported in different countries worldwide [25]. Our study found 21 types of herbal plants consumed among Thai patients with T2DM, with pandan leaf (*P.amaryllifolius*) being the most commonly used plant, followed by bitter gourd (*M. charantia*), country mallow (*A. indicum*), yanang leaf (*T. triandra*), and lingzhi mushroom (*G. lucidum*) (Table 2). It is important to note that these 21 plants were the plants the patients consumed at the highest frequently and continuously for six months. Indeed, plants in which the patients used a trace frequency in combination were not counted in our surveys. Among the 21 plants, pandan leaves, bitter gourds, moringa (*M. oleifera*), and passion fruit (*P. edulis*) were previously reported to show promising glucose-lowering activities in healthy individuals and patients with diabetes.

In contrast, the rest of them lacked scientific-based or systematic reports [7, 9, 38, 39]. Bitter gourd is the most commonly used plant among patients with T2DM in Malaysia [38] and has been used in many countries, including Tanzania, China, and India [40]. Consistent with a previous study [35], we observed that the majority of users (70.5%) chose a specific type of plant for their glycemic control based on the recommendations of relatives or friends (Figure 2). Access to scientific, evidence-based, and proven information on herbal medicine use for T2DM management directly by the patients in this study might be limited because of different factors, including their intrinsically low educational levels and monthly incomes or residence in rural areas, where some infrastructure, such as internet access, was not available. It has been found that 64.4% of Thai patients are reluctant to discuss their herbal medicine use with healthcare professionals [29]. Moreover, the beliefs and attitudes of healthcare staff towards CAM use, including herbal medicine, have been reported to vary from skepticism to conviction [18]. Therefore, healthcare professionals must be open-minded toward different T2DM therapies their patients may use and help them make safe choices of herbal plants for their glycemic control.

Not all herbal plants consumed by the patients maintained good glycemic control (HbA1c level <7%). All patients who consumed herbal plants in this study consumed them with their prescribed antidiabetic drugs. Their prescribed drugs could be divided into three patterns (500 mg/day metformin, 1000 mg/day metformin, and 2000 mg/day metformin plus 30 mg/day pioglitazone). Despite no evaluation by the Morisky Medication Adherence Scale [41], high adherence to the medication among the patients with T2DM in this study could be assumed, as all the patients were required by the hospital's policy to bring their medication into the clinics during follow-up before the medication could be refilled. A good glycemic control status was associated only with using herbal medicine combined with 500 mg/day of metformin (Table 3). The patients who consumed herbal plants with 500 mg/day of metformin were 2.92 times more likely to have good glycemic control than those who relied solely on 500 mg/day of metformin (adjusted OR = 2.921, 95% CI = 1.227–6.952, *p* = 0.015). Among the five most commonly used herbal plants, the use of bitter gourd with 500 mg/day of metformin exhibited a significant association with good glycemic control (adjusted OR = 8.33, 95% CI = 1.04–66.49, *p* = 0.046, Table 4). The major constituent of bitter gourd is charantin, which improves insulin sensitivity by increasing glucose uptake via glucose transporter-4 in skeletal muscle cells, resulting in cellular uptake and utilization of glucose [42]. This mechanistic finding was in line with an *in vivo* study where feeding mice with metformin (15 mg/kg) in combination with bitter gourd (300 mg/kg) showed synergistic hypoglycemic properties when compared to those that were fed with metformin or bitter ground alone [43]. However, the use of herbal medicine and higher doses of metformin was not found to be associated with good glycemic control in this study. This finding suggests that good glycemic control using herbal medicine is more likely influenced by the dosage of the prescribed antidiabetic drugs. A higher percentage of patients with good glycemic control (HbA1c<7%) was observed in the group of patients who had been prescribed 500 mg/day of metformin than in those who were prescribed higher doses or the maximally tolerated dose of metformin (2000 mg/day). It can be hypothesized that using herbal medicine for glycemic control would be effective only for patients with T2DM without metformin tolerance. Additionally, it was observed that patients who consumed >500 mg of metformin per day appeared to develop some diabetic complications, with a slightly higher percentage of them being non-users than those who consumed 500 mg/day of metformin. Our finding corresponded to the Indian study that patients with diabetes with comorbidities, such as vision impairment, cardiovascular disease, or neurological problems, were less likely to use CAM [44].

Our cross-sectional study is one of the very few studies that showed the use of herbal medicine and different dosages of antidiabetic agents for glycemic control management among patients with T2DM in district hospitals in Thailand. Herein, the initial hint of the usefulness of bitter gourd in improving glycemic control in T2DM patients who were prescribed 500 mg/day of metformin was suggested for the first time. However, prior to translating this glycemic control strategy into clinical practice at primary care clinics, continued research to clarify the mechanisms of action of major constituents (i.e., charantin) in bitter gourd, its long-term toxicity, the risk of developing herbal-drug interactions through drug-metabolizing enzymes (i.e., cytochrome P450s), and the efficacy of bitter gourd use by a randomized double-blinded clinical trial is required. The strength of this study is that the data were collected by trained healthcare professionals in four hospitals in different parts of Thailand. However, this study had certain limitations. First, it was a cross-sectional study; therefore, making causal inferences about the associations identified in this study was not appropriate. Second, despite being accepted as a legitimate scientific method, the data collected by some parts of the interview-based questionnaire (i.e., educational level and monthly income) were self-reported by the patients, which might be subject to social desirability bias and underreporting, which is more likely to occur. Third, the study was restricted to patients in district hospitals; therefore, the results may be limited in their generalization to patients visiting secondary or tertiary hospitals in Thailand or to the wider global population.

In summary, our findings showed associations between different variables and the use of herbal medicine among patients with T2DM who visited primary care clinics of district hospitals in Thailand, including age, duration of T2DM, BMI, physical activity levels, and dosages of the prescribed antidiabetic agents. The patients consumed 21 types of herbal plants for their glycemic control, whereas the individual patients decided to consume a particular plant based on recommendations from their relatives or friends. The association between herbal medicine usage and good glycemic control was found only when the herbal medicine was combined with 500 mg/day of metformin and not with higher doses of metformin. The specific type of plant contributing to this significant association was the bitter gourd. Data from our study could pave the way for further mechanistic studies to ensure the efficacy and safety of herbal medicine use in T2DM management, which would help improve the acceptability of herbal medicine usage by healthcare professionals and facilitate the integration of herbal medicine into national health systems, particularly in developing countries where health system coverage is limited.

## Declarations

### Author contribution statement

A. Prasopthum: Conceived and designed the experiments; Performed the experiments; Analyzed and interpreted the data; Wrote the paper.

T. Insawek: Performed the experiments.

P. Pouyfung: Conceived and designed the experiments; Performed the experiments; Analyzed and interpreted the data; Contributed reagents, materials, analysis tools or data; Wrote the paper.

### Funding statement

This work was supported by Walailak University, Thailand [Grant number: WU-IRG-62-037].

### Data availability statement

The data that has been used is confidential.

### Declaration of interest’s statement

The authors declare no conflict of interest.

### Additional information

Supplementary content related to this article has been published online at [URL].

## Appendix A Supplementary data

Questionaire_Plant_DM (final) Questionaire_Plant_DM (final)
