# Supplementary material for: Herbal medicine use in Thai patients with type 2 diabetes mellitus and its association with glycemic control: A cross-sectional evaluation
Source: Heliyon. 2022 Sep 28;8(10):e10790. doi: 10.1016/j.heliyon.2022.e10790 (PMC9535297; doi:10.1016/j.heliyon.2022.e10790)
Supplement: Questionaire_Plant_DM (final) [file mmc1.pdf]

ใบยินยอมเข้าร่วมโครงการวิจัย

ชื่อโครงการ การใช้พืชสมุนไพรของผู้ป่วยโรคเบาหวาน

หัวหน้าโครงการ ดร.อรุณา ประสพธรรม และ ฐิติพันธ์ อินทร์เสวก

ที่ปรึกษาโครงการ ดร.พิสิษฐ์ พวยฟุ้ง

วัตถุประสงค์ของการวิจัย

1) เพื่อสำรวจ ชนิด ปริมาณ และ ความถี่ ของการใช้พืชสมุนไพรในผู้ป่วยเบาหวานที่มารับการรักษาที่โรงพยาบาลชุมชน
2) เพื่อศึกษาความสัมพันธ์ ระหว่าง การใช้พืชสมุนไพรต่อการรักษาระดับน้ำตาล (HbA1c < 7%) ในผู้ป่วยเบาหวานชนิดที่ 2 ที่มารับการรักษาที่โรงพยาบาลชุมชน

เหตุผลที่ผู้เข้าร่วมโครงการวิจัยได้รับเชิญ/คัดเลือก

เนื่องจากโครงการวิจัยมีความประสงค์เพื่อสำรวจ ชนิด ปริมาณ และ ความถี่ ของการใช้พืชสมุนไพรในผู้ป่วยเบาหวานที่มารับการรักษาที่โรงพยาบาลชุมชน และเพื่อศึกษาความสัมพันธ์ ระหว่าง การใช้พืชสมุนไพรต่อการรักษาระดับน้ำตาล (HbA1c < 7%) ในผู้ป่วยเบาหวานชนิดที่ 2 ที่มารับการรักษาที่โรงพยาบาลชุมชนซึ่งผู้ที่ได้รับการรับเชิญมีคุณสมบัติที่ตรงกับกลุ่มตัวอย่างที่ต้องการศึกษาโดยจะต้องเป็นบุคคลที่มีความสมัครใจและยินดีตอบแบบสอบถามเพื่อนำข้อมูลที่ได้ไปวิเคราะห์ผล ตามวัตถุประสงค์ของโครงการวิจัย จำนวนผู้เข้าร่วมโครงการวิจัยทั้งหมดมี 375 คน ระยะเวลาที่ใช้ในการเก็บข้อมูล 1 ปี ตั้งแต่วันที่ 11 ตุลาคม 2561 - วันที่ 10 ตุลาคม 2562 ผลการศึกษาจะนำไปสู่การให้ความรู้กับผู้ป่วยในการเลือกใช้พืชสมุนไพรกับการควบคุมระดับน้ำตาลในผู้ป่วยเบาหวานชนิดที่ 2 ร่วมกับยาแผนปัจจุบัน ถ้าท่านยินยอมเข้าร่วมโครงการวิจัย ท่านจะถูกสัมภาษณ์จากเจ้าหน้าที่ โดยใช้แบบสอบถามในการเก็บข้อมูล

สิ่งที่ผู้วิจัยจะขอให้ผู้เข้าร่วมโครงการวิจัยปฏิบัติ

ขอให้ท่านตอบแบบสอบถาม 1 ครั้ง ใช้เวลาประมาณ 15-20 นาที

การตอบแบบสอบ แบ่งออกเป็น 3 ส่วนดังนี้

ส่วนที่ 1 ข้อมูลส่วนบุคคล (Part 1: Personal Sociodemographic Data)

ส่วนที่ 2 ข้อมูลการใช้พืชสมุนไพร (Part 2: Herbal medicine usage)

ส่วนที่ 3 ข้อมูลส่วนนี้เป็นข้อมูลที่ตรวจสอบจากเวชระเบียนของผู้ป่วย

(Part 3: Patient's specific information from the medical records)

ความเสี่ยงที่อาจจะเกิดเมื่อเข้าร่วมโครงการวิจัย

ความเสี่ยงหรือความไม่สบายที่อาจเกิดขึ้นกับอาสาสมัครที่เข้าร่วมโครงการครั้งนี้มีความเสี่ยงน้อยมาก ไม่เกินความเสี่ยงที่ผู้เข้าร่วมมีอยู่ในการดำเนินชีวิตประจำวัน ซึ่งอาจทำให้ผู้เข้าร่วมโครงการวิจัยเสียเวลาประมาณ 15 – 20 นาที อาจทำให้ผู้เข้าร่วมโครงการเสียเวลาในการตอบ และอาจมีบางข้อคำถามที่รบกวนจิตใจผู้ถูกสัมภาษณ์ทำให้ไม่สบายใจ

วิธีการเก็บข้อมูลของกลุ่มตัวอย่างเป็นความลับ

ข้อมูลส่วนตัวของท่านจะถูกเก็บรวบรวมไว้ตู้เอกสารที่มีระบบรักษาความลับและปลอดภัยซึ่งผู้ไม่เกี่ยวข้องไม่สามารถเข้าถึงได้ โดยผู้วิจัยจะเปิดเผยข้อมูลเพื่อประโยชน์ในทางวิชาการโดยไม่ระบุชื่อ ข้อมูลส่วนตัวของผู้เข้าร่วมโครงการวิจัยจะถูกเก็บรักษาไว้ไม่เปิดเผยต่อสาธารณะเป็นรายบุคคล

ท่านมีสิทธิ์ถอนตัวออกจากโครงการวิจัยได้ตลอดเวลา การตัดสินใจของท่านจะไม่มีผลต่อการรักษาในอนาคต การดูแลอื่นใดก็ตาม หรือสูญเสียประโยชน์ใด ๆ หากท่านไม่ต้องการเข้าร่วมการศึกษาหรือต้องการหยุดการศึกษา ณ เวลาใดก็ตาม โดยข้อมูลที่ท่านได้เปิดเผยแล้วจะยังเก็บรักษาไว้เป็นความลับเฉพาะ

หากท่านได้รับการปฏิบัติที่ไม่ตรงตามที่ได้ระบุไว้ในเอกสารชี้แจงนี้ ท่านสามารถแจ้งให้คณะกรรมการจริยธรรมการวิจัยในมนุษย์ทราบได้ที่ สำนักงานคณะกรรมการจริยธรรมการวิจัยในมนุษย์ อาคารวิจัยวิทยาการสุขภาพ เลขที่ 222 ต.ไทยบุรี อ.ท่าศาลา จ.นครศรีธรรมราช โทร 075-672554-3 โทรสาร 075-672551 (ในวันเวลาราชการ)

ลงชื่อ…………….......……………….............……...……ผู้เข้าร่วมโครงการวิจัย

(…………….......……………….............……...……)

ลงชื่อ……………………………..............……...…………พยาน

(……………………………..............……...…………)

ลงชื่อ……………………………..............……...…………พยาน

(……………………………..............……...…………)

วันที่…………….เดือน……….........………..พ.ศ…………......

Informed Consent Form

Title of project: The use of herbal medicine in type 2 diabetes

Investigators: Dr. Aruna Prasothum, Thitipan Insawek

Principal Investigator: Dr. Phisit Pouyfung

Purpose of the research:

To assess the prevalence of herbal medicine use in Thai patients with type 2 diabetes mellitus (T2DM) and its association with glycemic control.

What will you be asked to do for your participation in this research?

We would like to invite you to participate in our research study on "The use of herbal plants in type 2 diabetes", which seeks to identify herbal medicine plants usage among type 2 diabetes in primary healthcare hospitals. We urge you to discuss any questions about this study with our trained healthcare professionals. If you decide to participate, you must sign this form to show that you are willing to participate. Approximately at least 375 people will take part in this research and the data will be collected from 11 October 2018 to 10 October 2019. The results of this study will reveal the association between herbal medicine used among type 2 diabetes and glycemic control, which might help to improve the acceptability of healthcare professionals towards complementary and alternative medicine specifically herbal medicine for diabetes management together with the conventional medicine. If you agree to participate in our research program, you will be interviewed by our trained healthcare staff using a questionnaire, which will take you about 20-30 minutes.

A questionnaire used in this study contains 3 main following parts:
Part 1: personal sociodemographic data
Part 2: herbal medicine usage
Part 3: patient's specific information from the medical records

Risks and discomforts

There is no risk from participating in this study. Your participation is voluntary; thus, you may withdraw from this study at any time. There will be no penalty or any effect if you decide to refuse to participate.

Data privacy protection

You will not be identified by name, identification number, address, or phone number for the research records except HN number associated with hemoglobin A1c value. The collected information of all participants will be identified by a random code number and will be kept in a locked file in Dr. Phisit Pouyfung's office. None of your answers will be publicly revealed. All your responses and information will only be used for the purpose of this study. All identifiable information will be destroyed after the completion of the study.

This research has been received ethical review and been approved by the Ethics Committee in Human Research of Walailak University (ECHR-WU), Thailand. If you have questions regarding your participation, data collection process in this research, or your rights as a participant in the study, please do not hesitate to contact:

1. Dr. Phisit Pouyfung, School of Public Health, Walailak University
   Telephone no.: +66(0)75-672754.

2. Walailak University Ethics Committee in Human Research of Walailak University
   Telephone no.: +66(0)75 672595
   E-mail address: wu.wuec@gmail.com

______________________	______________________
Signature of participant	Date
______________________	______________________
Signature of witness	Date
______________________	______________________
Signature of witness	Date

Questionnaire

การใช้พืชสมุนไพรของผู้ป่วยโรคเบาหวาน (The use of herbal medicine in type 2 diabetes**)**

คำชี้แจง ขอให้บุคลากรทางการแพทย์ผู้ทำการสัมภาษณ์ทำเครื่องหมาย ✓และเติมคำในช่องว่างที่ตรงกับข้อมูลตามความเป็นจริงของผู้ป่วยที่เข้ารับการสัมภาษณ์

(Please a trained healthcare professional check the appropriate box and fill out the patient information into the corresponding blank areas)

แบบสอบถามประกอบด้วย 3 ส่วน คือ

(This form will collect the patient data, which can be largely categorized into three main parts)

ส่วนที่ 1 ข้อมูลส่วนบุคคล จำนวน 11 ข้อ (Personal sociodemographic data: 11 Questions)

ส่วนที่ 2 ข้อมูลการใช้พืชสมุนไพร (Herbal plant usage data)

ส่วนที่ 3 ข้อมูลที่ตรวจสอบจากเวชระเบียนของผู้วิจัย (Patient's specific information)

ส่วนที่ 1 ข้อมูลส่วนบุคคล (Part 1: Personal Sociodemographic Data)

1. เพศ (Gender) ☐ ชาย (Male) ☐ หญิง (Female)

2. อายุ (Age) ……...…...ปี (Year)

3. น้ำหนัก (Weight) …………..กิโลกรัม (Kg)

4. ส่วนสูง (Height) …………..เซนติเมตร (cm)

5. สถานภาพสมรส (Marital status) ☐ โสด (Single) ☐ แต่งงานแล้ว (Married) ☐ หย่าร้าง (divorced)

6. ระดับการศึกษา (Educational levels)

   ☐ ประถมศึกษา 1-6 (Primary School)

   ☐ มัธยมศึกษา 1-6 (Secondary school)

   ☐ อุดมศึกษา (University)

7. อาชีพ (Occupations)

   ☐ งานบ้าน/ว่างงาน (Unemployed)

   ☐ ค้าขาย (Grocer)

   ☐ เกษตรกรรม (Agriculturist)

   ☐ รับราชการ/ รัฐวิสาหกิจ (Government official)

   ☐ รับจ้างทั่วไป/ประมง (Fisherman)

   ☐ อื่น ๆ (Others) ระบุ (please specify)……………………….............

8. รายได้เฉลี่ยต่อเดือนของครอบครัว (Monthly income)……………………………..บาท (Baht)

9. ระยะเวลาที่ป่วยเป็นโรคเบาหวานนับจากวันที่ถูกวินิจฉัย (Duration of having T2DM from the first diagnosis)

   …………………. ปี (year) …………………. เดือน (month)

10. ท่านออกกำลังกายหรือไม่ (Do you exercise?)

   ☐ ไม่ออกกำลังกาย (No)

   ☐ ออกกำลังกาย (Yes)

      ☐ ออกกำลังกายระดับหนักมาก (Vigorous intensity)

      เช่น วิ่งจ๊อกกิ้ง จนเหนื่อยมากพูดไม่ได้ วันละอย่างน้อย 20 นาที สัปดาห์ละอย่างน้อย 3 วัน

      (For instance, any forms of aerobic exercise in which the patient cannot speak while performing the activity and not less than 20 min per day and 3 days a week)

      ☐ ระดับหนักพอควร (Moderate intensity)

      ซึ่งทราบจากการที่เหนื่อยจนร้องเพลงไม่ได้ แต่ยังพอพูดได้ อย่างน้อยวันละ 30 นาที สัปดาห์ละอย่างน้อย 5 วัน

(For instance, any forms of activities in which the patient cannot sing a song but can still speak while performing the activity and not less than 30 min per day and 5 days a week)

☐ ในระดับเบา (Light intensity)
เช่น การทำงานบ้าน ล้างจาน อาบน้ำ เดินขึ้นลงบันได
(For instance, any forms of daily activities including walking in the offices/buildings or in shopping stores, dish washing, laundry, driving, and etc.)

11 ข้อมูลการรับประทานอาหารย้อนหลัง 24 ชั่วโมง (24-hour dietary recall)
*The healthcare professionals who perform the interview should use the provided real examples of Thai meal sets including stream rice, boiled noodles, grilled pork, and salad so that the patient can see and use them to estimate how much the amount of food they have consumed for each meal.

- ชื่ออาหารเช้า (Breakfast name) ……………………………………………………….…………………
  - ปริมาณคาร์โบไฮเดรต (Estimated Amount of Carbohydrate) ....กรัม (gram)
  - ปริมาณโปรตีน (Estimated Amount of Protein) ....กรัม (gram)
  - ปริมาณไขมัน (Estimated Amount of Fat) ....กรัม (gram)

- อาหารกลางวัน (Lunch name) …………………………….……………………………………………..
  - ปริมาณคาร์โบไฮเดรต (Estimated Amount of Carbohydrate) ....กรัม (gram)
  - ปริมาณโปรตีน (Estimated Amount of Protein) ....กรัม (gram)
  - ปริมาณไขมัน (Estimated Amount of Fat) ....กรัม (gram)

- อาหารเย็น (Diner name) …………………………………………………………………………………
  - ปริมาณคาร์โบไฮเดรต (Estimated Amount of Carbohydrate) ....กรัม (gram)
  - ปริมาณโปรตีน (Estimated Amount of Protein) ....กรัม (gram)
  - ปริมาณไขมัน (Estimated Amount of Fat) ....กรัม (gram)

- น้ำหวาน (Sugar-sweetened beverage) …………………………… ปริมาณ…………… (liter)
  จำนวนครั้งที่ดื่ม (serving)………………………………………………

ส่วนที่ 2 ข้อมูลการใช้พืชสมุนไพร (Part 2: Herbal medicine usage)

ชื่อสมุนไพร (Thai common name of the herbal plant used by the patient)

…………………………………………………………………………..…………………………………….

ส่วนใดที่นำมาใช้ (Part of use)

…………..………………………………………………………………………………………..………

รูปแบบที่ใช้ (Form of supplement i.e., Tea, Beverage, Capsule, etc.)

…………………………………………………………………………………………………...……………

ระยะเวลาในการกิน (How often do the patient use the herbal plant per day?)

…………………………………………………...……………………………………………………………

กินแล้วมีอาการข้างเคียงอย่างไร (Adverse side effect after consumption)

……………………………………………………………………………………………..…………………….

รู้ได้อย่างไรว่าพืชที่รับประทานสามารถใช้รักษาโรคเบาหวาน (Sources of the information whereby the patients perceive that the herbal plant can be used for lowering blood sugar)

……

ส่วนที่ 3 ข้อมูลส่วนนี้เป็นข้อมูลที่ตรวจสอบจากเวชระเบียนของผู้ป่วย

(Part 3: Patient's specific information from the medical records)

เวชระเบียนของผู้ป่วยจากโรงพยาบาล (Specify the name of the hospital where the data were collected)

……..

HN ……………………………………………………………………………………………………….

(Specify HN to verify that the data is not fabricated, but rather collected from the medical record of the patient. However, this data here which show patient identification will be securely stored, not be exposed to public, and only used for research purpose only according to the ethic regulated by Human Research Ethics Committee of Walailak University, Thailand.)

1.ฮีโมโกลบินเอวันซี (HbA1C)

วันที่อ่านผลเลือด (Date of measurement)	%	ทำเครื่องหมายถูกเมื่อเช็คอีกครั้งกับ Medical record แล้ว (Check the box when the data is matched with the patient's medical record)
		☐

2.การรักษาโรคเบาหวานปัจจุบันของผู้ป่วย (The prescribed oral anti-diabetic agents)

1. ชื่อยา (Name of prescription) ....
   ขนาดเม็ด (Dosage)…………………………………………………………………….…….(มก/mg)
   ขนาดยาต่อวัน (dispensing daily quantity)………………………………………………..(มก /mg)
   วิธีการใช้จำนวน (Duration of use) ………………………………………………………(ครั้ง/วัน)
   ☐ Tick when the data is matched with the patient's medical record

2. ชื่อยา (Name of prescription) ....
   ขนาดเม็ด (Dosage)…………………………………………………………………….…….(มก/mg)
   ขนาดยาต่อวัน (dispensing daily quantity)………………………………………………..(มก /mg)
   วิธีการใช้จำนวน (Duration of use) ………………………………………………………(ครั้ง/วัน)
   ☐ Tick when the data is matched with the patient's medical record

3. ชื่อยา (Name of prescription) ....

ขนาดเม็ด (Dosage)…………………………………………………………………….…….(มก/mg)

ขนาดยาต่อวัน (dispensing daily quantity)………………………………………………..(มก /mg)

วิธีการใช้จำนวน (Duration of use) ……………………………………………………….(ครั้ง/วัน)

☐ Tick when the data is matched with the patient’s medical record

Other comments:

……

……………………………………………………………………………………….……………...

……

……

……

ลงชื่อ (Sign) …………………………………………………………………………..

ผู้บันทึกแบบสอบถาม …………………………………………………………………………

(A healthcare professional who fills out the information)

ตำแหน่ง (position) ……………………………………………………………………………….

วันที่บันทึกข้อมูล (Date when the data being collected) …………………...…………………….
